# Methylated markers accurately distinguish primary central nervous system lymphomas (PCNSL) from other CNS tumors

**DOI:** 10.1186/s13148-021-01091-9

**Published:** 2021-05-05

**Authors:** Bradley M. Downs, Wanjun Ding, Leslie M. Cope, Christopher B. Umbricht, Wenge Li, Huihua He, Xiaokang Ke, Matthias Holdhoff, Chetan Bettegowda, Weiping Tao, Saraswati Sukumar

**Affiliations:** 1 Departments of Oncology, Johns Hopkins University School of Medicine, Baltimore, MD 21231 USA; 2 Department of Oncology, Renmin Hospital of Wuhan University, Wuhan, 430060 Hubei People’s Republic of China; 3 Departments of Surgery, Johns Hopkins University School of Medicine, Baltimore, MD 21231 USA; 4 Department of Pathology, Renmin Hospital of Wuhan University, Wuhan, 430060 Hubei People’s Republic of China

**Keywords:** DNA methylation, Epigenetic biomarkers, TAM-MSP, Primary central nervous system lymphoma, Liquid biopsy, Circulating tumor DNA

## Abstract

**Background:**

Definitive diagnosis of primary central nervous system lymphoma (PCNSL) requires invasive surgical brain biopsy, causing treatment delays. In this paper, we identified and validated tumor-specific markers that can distinguish PCNSL from other CNS tumors in tissues. In a pilot study, we tested these newly identified markers in plasma.

**Results:**

The Methylation Outlier Detector program was used to identify markers in TCGA dataset of 48 diffuse large B-cell lymphoma (DLBCL) and 656 glioblastomas and lower-grade gliomas. Eight methylated markers clearly distinguished DLBCL from gliomas. Marker performance was verified (ROC-AUC of ≥ 0.989) in samples from several GEO datasets (95 PCNSL; 2112 other primary CNS tumors of 11 types). Next, we developed a novel, efficient assay called Tailed Amplicon Multiplexed-Methylation-Specific PCR (TAM-MSP), which uses two of the methylation markers, cg0504 and *SCG3* triplexed with *ACTB*. FFPE tissue sections (25 cases each) of PCNSL and eight types of other primary CNS tumors were analyzed using TAM-MSP. TAM-MSP distinguished PCNSL from the other primary CNS tumors with 100% accuracy (AUC = 1.00, 95% CI 0.95–1.00, *P* < 0.001). The TAM-MSP assay also detected as few as 5 copies of fully methylated plasma DNA spiked into 0.5 ml of healthy plasma. In a pilot study of plasma from 15 PCNSL, 5 other CNS tumors and 6 healthy individuals, methylation in cg0504 and *SCG3* was detectable in 3/15 PCNSL samples (20%).

**Conclusion:**

The Methylation Outlier Detector program identified methylated markers that distinguish PCNSL from other CNS tumors with accuracy. The high level of accuracy achieved by these markers was validated in tissues by a novel method, TAM-MSP. These studies lay a strong foundation for a liquid biopsy-based test to detect PCNSL-specific circulating tumor DNA.

**Supplementary Information:**

The online version contains supplementary material available at 10.1186/s13148-021-01091-9.

## Background

Primary central nervous system lymphoma (PCNSL) is a rare but aggressive form of extranodal non-Hodgkin lymphoma (NHL) limited to the brain, spinal cord, leptomeninges and eyes [1]. More than 95% of PCNSL are of the diffuse large B-cell lymphoma subtype (DLBCL) [2], which are further divided into two major molecular subtypes, germinal center B-cell (GCB) and activated B-cell (ABC) DLBCL [3–5], based on their cell of origin. The majority of PCNSL show a gene expression profile typical of ABC-DLBCL [6]. With an incidence of 0.44 per 100,000, PCNSL accounts for approximately 2% of all primary central nervous system (PCNS) tumors [7]. Since 2000, there has been an increase in the overall incidence of PCNSL, especially in the elderly, with a median age at diagnosis of 65 years [7], and the highest incidence occurs in the oldest age groups [8]. Effective, and potentially curative treatment options exist [9, 10]. However, the overall prognosis of PCNSL is poor, with the 5- and 10-year survival rates for PCNSL at 29.9% and 22.2%, respectively [7].

The current diagnosis of PCNSL is based on imaging and analysis of the cerebrospinal fluid (CSF) for abnormal cells. Both these methods are often challenging as standard imaging is non-specific, and CSF analysis is, most often, inconclusive [11]. In most cases, neurosurgical biopsy and cytological analysis are required to make the diagnosis of PCNSL. However, tissue biopsies are invasive, can be technically challenging and often yield inadequate tissue for molecular profiling [12–14]. In addition, patients frequently receive steroids during their workup which lowers the sensitivity of a histopathological diagnosis [14]. Complex workup can lead to significant delays in starting therapy, which can lead to irreversible neurological defects. Since there are highly effective and potentially curative treatment options for PCNSL and as the treatment for PCNSL is very different from that of other primary cancers of the CNS, accurate and timely diagnosis is of great importance.

Disease-specific circulating biomarkers in the CSF or in blood may circumvent the limitation of standard diagnostics. A prerequisite for such an approach is availability of candidate markers with high specificity for the disease, as well as techniques to detect these markers with high sensitivity. Previous studies have shown that detection of tumor-specific cell-free DNA in CSF of primary brain cancers, including PCNSL, is feasible using next-generation sequencing-based techniques [10, 15]. Other markers, including proteins and micro-RNA, have been studied, but their specificity for PCNSL is often limited [11].

In recent years, DNA methylation markers have been tested extensively for tumor diagnosis, therapeutic monitoring and prognosis of long-term outcome [16, 17]. Due to the rarity of PCNSL, there are only a few reports on DNA methylation markers in this disease using archival tissue [18–21]. Two papers on consecutive DNA methylation array studies by the same group reported that they found no significant differences between PCNSL and non-CNS DLBCL [18, 21], and similar alterations were detected in PCNSL and DLBCL compared to normal blood cells. Neither paper took other CNS tumors into consideration.

In this study, we examined several large, public 450K methylation array datasets obtained from TCGA, the Gene Expression Omnibus (GEO) and a Bioconductor data library to select and test markers of PCNSL. We identified DNA methylation markers that have the ability to distinguish PCNSL from other malignant PCNS tumors and tested the markers in a sample set of archival formalin fixed, paraffin embedded (FFPE) samples from Wuhan using the newly developed quantitative laboratory assay, TAM-MSP. We were able to achieve 100% accuracy in distinguishing between PCNSL and eight other types of PCNS tumors. This new assay was also able to detect as few as 5 copies of methylated DNA spiked into 0.5 ml of healthy plasma. In a small pilot liquid biopsy study using plasma, we show that the TAM-MSP assay could detect methylated DNA in 3 out of 15 newly diagnosed PCNSL patients. From this study, we suspect that measuring methylated circulating tumor DNA (ctDNA) from liquid biopsies, such as CNS, may be needed to accurately distinguish a large percentage of PCNSL patients for patients with other CNS tumors.

## Results

### Identification and selection of methylated markers in TCGA

**Fig. 1:**
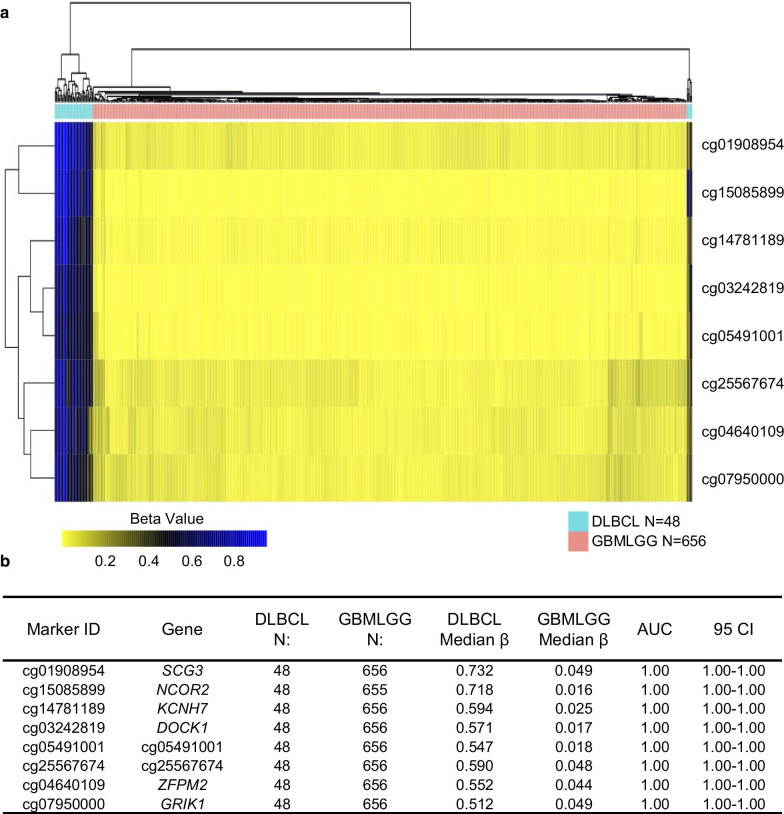
Identification of methylation markers by the Methylation Outlier Detector program in TCGA dataset. **a** The Methylation Outlier Detector program in TCGA dataset identified 8 potential methylation markers. A hierarchical cluster heatmap displaying the methylation levels of the 8 identified markers showed separation of all of the DLBCL from GBMLGG samples. **b** The table presents the methylation characteristics and performance of each of the 8 markers for its ability to distinguish DLBCL from GBMLGG samples. *N* number of samples, *AUC* area under the curve, *CI* confidence interval, *DLBCL* lymphoid neoplasm diffuse large B-cell lymphoma, *GBMLGG* glioblastoma multiformae and lower-grade gliomas

Since it has been previously shown that the DNA methylome of the lymphoid neoplasm, diffuse large B-cell lymphoma (DLBCL), is indistinguishable from that of PCNSL [18, 21], we first searched for DNA methylation markers for PCNSL in TCGA data by comparing DLBCL (*N* = 48) with other CNS tumors, glioblastoma and lower-grade gliomas (GBM and LGG, *N* = 656). Using our Methylation Outlier Detector algorithm, we identified 8 markers (cg01908954-*SCG3*, cg15085899-*NCOR2*, cg14781189-*KCNH7*, cg03242819-*DOCK1*, cg05491001-cg054, cg25567674-cg255 cg04640109-*ZFPM2* and cg07950000-*GRIK1*) (Additional file 5: Table S1) that were highly methylated (*β* value greater than 0.20) in all 48 DLBCL samples, and had a *β* value less than 0.15 in all 656 of the GBM and LGG samples. The heatmap (Fig. 1a) shows clear segregation and differential methylation in lymphomas, in contrast to brain tumors, and the high ROC-AUC achieved by these markers is shown in Fig. 1b.

### Verification of methylated markers in GEO datasets

**Fig. 2:**
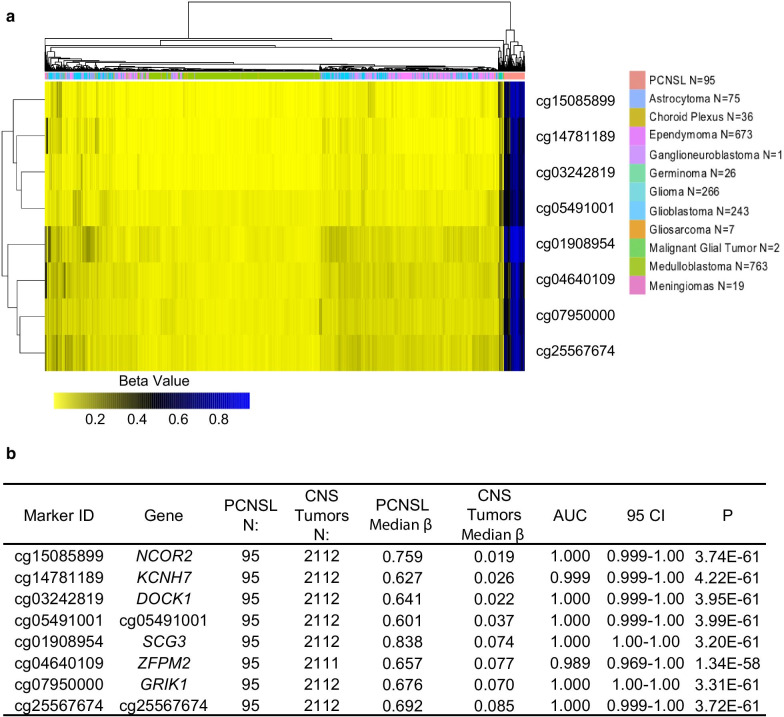
Verification of the 8 methylated markers in the GEO datasets. **a** A hierarchical cluster heatmap shows the methylation intensity of the 8 markers in databases of PCNSL and 11 other CNS tumors downloaded from the GEO database. **b** The table presents the methylation characteristics and performance of each of the 8 markers to distinguish PCNSL from the 11 other CNS tumor samples. *N* number of samples, *AUC* area under the curve, *CI* confidence interval, *P* Mann–Whitney statistics, *PCNSL* primary central nervous system diffuse large B-cell lymphoma, *CNS* central nervous system

The 8 candidate markers were then verified in independent, publicly available data compiled from several Gene Expression Omnibus submissions. In addition to 95 PCNSL samples, these datasets include 2112 CNS tumors representing 11 different brain tumor types. As shown in the heatmap, the same eight markers strongly distinguished PCNSL from the other brain tumors (Fig. 2a). Each of the 8 markers could accurately distinguish between the PCNSL samples and the 11 other CNS tumor types (AUC ranging between 0.989 and 1.00, *P* < 0.001) (Fig. 2b). The median *β* values of the 8 markers in the PCNSL ranged between 0.601 and 0.838 compared to the other CNS tumors where the median β values ranged between 0.019 and 0.085 (Fig. 2b).

To investigate whether the 8 identified markers are specific to B-cell lymphomas, and are not methylated in normal B cells, each marker was assessed in databases of hematopoietic stem cells (*N* = 8), B-cell precursors (*N* = 22), B-cell (*N* = 56), PCNSL (*N* = 95), acute lymphoblastic leukemia (ALL) (*N* = 175) and chronic lymphocytic leukemia (CLL) (*N* = 196) (Additional file 1: Fig. S1). All 8 markers showed significantly higher levels of DNA methylation (*P* < 0.001, Mann–Whitney) in the PCNSL tissues (median *β* values, range 0.601–0.838) compared to normal B-cells (median *β* values, range 0.164–0.364). We also observed that methylation levels in PCNSL for the 8 markers showed, as expected, overlap with CLL and ALL, but showed tighter clusters and had less overlapping methylation levels with the B-cells than ALL and CLL (Additional file 1: Fig. S1). We found that 5 of the 8 markers (cg054, *SCG3*, *KCNH7*, *ZFPM2* and *GRIK1*) showed distinctly higher levels of methylation in PCNSL, ALL and CLL in comparison with the hematopoietic stem cells, B-cell precursors and B-cells. Interestingly, *NCOR2* methylation levels for B-cell precursors, ALL and CLL were all greater than hematopoietic stem cells, B-cells and PCNSL.

#### Additional considerations for PCNSL markers detection in blood

Markers that identify PCNSL and can distinguish this disease from other CNS tumors are needed, but this property alone is not sufficient for markers to be useful for analysis of blood, where DNA shed by other types of cells is present in abundance. To investigate the methylation levels of the eight markers in DNA commonly shed by other cells in blood, we analyzed the GEO datasets from PCNSL (*N* = 95), normal brain (*N* = 50), whole blood (*N* = 656), buffy coat (*N* = 35), cell-free DNA (cfDNA) from pooled healthy plasma (*N* = 4) and cfDNA from plasma from healthy individuals (*N* = 4) (Additional file 2: Fig. S2). We found that methylation levels for the eight markers were significantly higher in PCNSL than in all datasets derived from normal brain or components of blood (*P* < 0.001, Mann–Whitney) (Additional file 2: Fig. S2).

We had observed by analysis of the GEO databases (Additional file 2: Fig. S2) that the methylation level for each of the 8 markers was lower in pooled plasma than in the buffy coat or whole blood samples (*P* < 0.001, Mann–Whitney), raising the possibility that the buffy coat is the source of background methylation that is observed in whole blood. We also observed that the individual plasma samples had higher levels of methylation than the pooled plasma samples and did not show significantly differential methylation in comparison with whole blood in 5 of the 8 markers (cg054, *SCG3*, *DOCK1*, cg255 and *ZFPM2*). It should be noted that the difference between pooled plasma and individual plasma may not be reliable since it is based on a small sample size (*N* = 4) in both groups.

#### Analytical validation of the PCNSL markers

Our in silico analysis indicated that even one marker would be sufficient to distinguish between PCNSL and other CNS neoplasms with accuracy. However, a limited number of PCNSL exist in these datasets and may not reflect the heterogeneity of all PCNSL. Our quantitative assays are also capable of analyzing up to 2 markers in the same qPCR. Therefore, we took the following steps to select two-marker panels (from the 8 markers identified in TCGA) with the best performance. We first calculated the cumulative methylation value for each of 28 possible combinations of two markers in the GEO dataset. Next, we compared the calculated AUC of the ROC for each of 28, two-marker panels. We found that 5 of the 28 (18%) two-marker combinations achieve 100% accuracy in distinguishing PCNSL samples from the other 11 CNS tumor types. The top five, two-marker panels were combinations of: *SCG3* and *DOCK1*, *SCG3* and cg054, *SCG3* and cg255, *DOCK1* and cg054, and *GRIK1* and cg255. In a second validation dataset (GSE90496) [22], comprised of 13 PCNSL and 2,788 other tumors of the CNS and normal CNS tissues, we found that the cumulative methylation value for each of the top five two-marker panels had an AUC that ranged between 1.000; 95% CI 0.999–1.000 and 1.000; 95% CI 1.000–1.000; Mann–Whitney *P* < 0.001.

To further select the best two markers, we developed primers and probes for 5 of the 8 markers identified from the methylome data (Additional file 6: Table S2) and tested amplification efficiency using fully methylated DNA and fully unmethylated DNA using our laboratory assay, quantitative multiplexed-methylation-specific PCR (QM-MSP) [23, 24]. To enable development of a highly sensitive and specific assay, we screened each of the markers to select those with the highest amplification efficiency. We found that the markers, *SCG3* and cg054, amplified with the highest efficiency (earliest Cts) and specificity in the unmethylated control, compared to the other 3 markers. Combining the two attributes of 100% accuracy in the GEO dataset (Fig. 2) and the high efficiency of amplification in the QM-MSP assay, *SCG3* and cg054 were selected as the optimal two-marker panel.

**Fig. 3:**
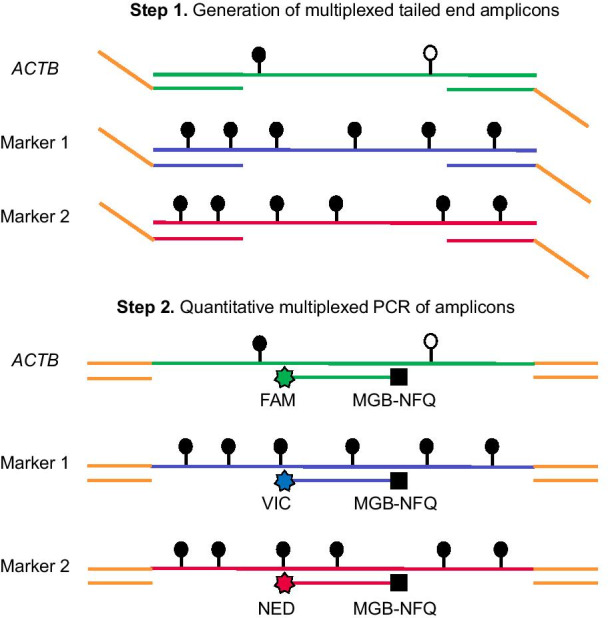
Schema of the TAM-MSP method. *The first step* of TAM-MSP amplifies two markers and actin control (*ACTB*) control using a single aliquot of DNA in one well, with primers located within the CpG-rich region of interest for each methylation marker, and therefore includes CpG dinucleotides in its sequence. The 5′ end of the forward and reverse primers for the two methylation markers and *ACTB* have the same synthetic tails. *In the second step* of TAM-MSP, primers that are complimentary to the synthetic tails are used, along with marker-specific TaqMan probes, each with one of three indicated fluorescent tags. All three markers are amplified in a single real-time PCR. Methylation in each marker is quantified through interpolation on a historic standard curve and is expressed as percent cumulative methylation. Open circles: unmethylated CpG; closed circles: methylated CpG

Next, we developed and tested a new simplified assay, TAM-MSP, tailored for measuring ctDNA methylation from plasma in multiple markers (schema in Fig. 3). The two-marker panel consisting of *SCG3* and cg054 along with *ACTB* used as a loading control was tested in a quantitative methylation-specific PCR assay that could be conducted in a two consecutive triplexed qPCRs (Fig. 3).

In the first amplification step, in a single reaction, using one aliquot of DNA, 5′ synthetic tails in the three sets of forward and revisers primers were incorporated into the amplicons. In the second amplification step, each marker was amplified using primers that were complementary to the 5′ synthetic tails, along with a marker-specific probe, tagged with one of the three different fluorophores and a common quencher (Additional file 7: Table S3).

The TAM-MSP assay showed linearity (*R*^2^ > 0.90) with DNA samples containing 100–3% methylation and accurately measured as little as 63 pg of methylated DNA, which is the equivalent of 10 cells (Additional file 3: Fig. S3a and b).

**Fig. 4:**
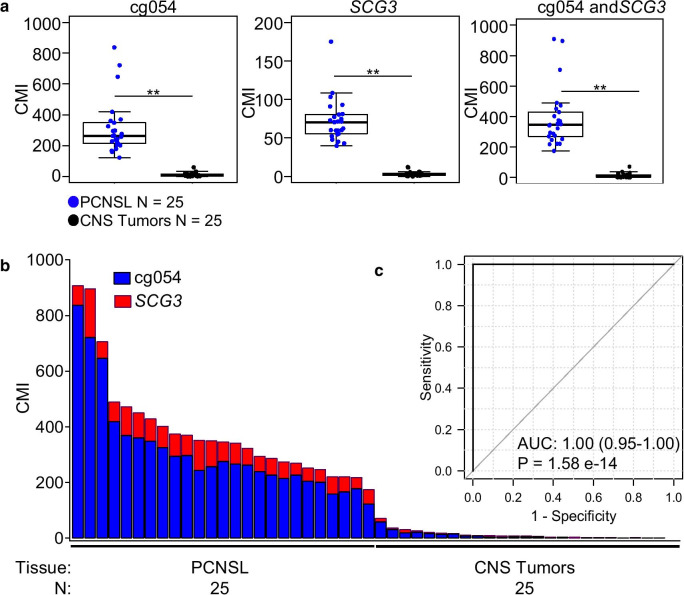
Performance of the two markers in the Wuhan clinical sample set using the TAM-MSP method. **a** The two markers, cg0504 and *SCG3*, assessed as a panel or assessed individually, achieved 100% accuracy in distinguishing PCNSL from the 8 other CNS tumors. **b** The histogram of cumulative methylation for the two-marker panel in each sample displays the contribution of the two markers in detecting PCNSL among the CNS tumors in the sample set. **c** Analysis of receiver operator characteristics (ROC, inset) shows that the two-marker panel performed with a high degree of accuracy with AUC of 1.00 (CI 0.95–1.00). **Mann–Whitney *P* < 0.001; *CMI* cumulative methylation index, *N* number of samples, *P* Mann–Whitney statistics, *AUC* area under the curve

Using TAM-MSP, we tested the two markers in the Wuhan clinical sample set of FFPE tissues of PCNSL (*N* = 25) and CNS tumors (*N* = 25) comprised of 8 different tumor types (Table 1). The sample with the highest methylation in other CNS tumors (*N* = 25) showed a CMI of 70.9 units, while the sample with the lowest methylation value in PCNSL (*N* = 25) showed a CMI of 174.4 units (a separation of more than 100 CMI units). TAM-MSP performed with a high level of accuracy, as shown in the box plots, singly and as a two-marker panel AUC of 1.00 (CI 0.95–1.00), Mann–Whitney *P* < 0.001 (Fig. 4a). As shown in the histogram (Fig. 4b) PCNSL tumors displayed high levels of cumulative methylation, while other CNS tumors showed low or no detectable levels of methylation in the two markers. Analysis of receiver operator characteristics (Fig. 4c) showed that the two-marker panel showed a high level of accuracy with AUC of 1.00 (CI 0.95–1.00, Mann–Whitney *P* = 1.58e−14). Using QM-MSP, we tested the same two markers, and additionally, the 3 other markers, which included *DOCK1*, *KRIK1* and *KCNH7*, that were out-performed by cg054 and *SCG3* for amplification efficiency. Each of the 5 markers independently (Additional file 4: Fig. S4a) and as a two-marker panels (Additional file 4: Fig. S4b) distinguished between PCNSL and other CNS tumors with accuracy (ROC-AUC = 1.00, 95% CI 0.95–1.00, Mann–Whitney *P* = 1.58e−14) (Additional file 4: Fig. S4c).

**Table 1:** Patient characteristics of the Wuhan clinical sample set

CNS tumors	Glioblastoma	Astrocytoma	Ependymoma	Germinoma	Medulloblastoma	Oligodendroglioma	Meningioma	Solitary fibrous tumor	Total	PCNSL	Plasma glioblastoma and glioma	Plasma PCNSL
*N* =	6	4	2	2	3	4	3	1	25	25	5	15
Age												
Median	46	36	36	21	12	44	47	32	39	58	48	64
Range	17–53	35–41	3–68	9–32	6–26	38–50	45–66	–	3–68	35–71	14–66	46–84
Gender												
Male	4	2	2	2	1	2	2	–	15	11	4	9
Female	2	2	–	–	2	2	1	1	10	14	1	6
WHO grade												
I	–	–	–	–	–	–	–	–	–	–	–	–
II	–	1	–	–	–	–	–	–	1	–	–	–
III	–	3	2	–	–	4	3	1	13	–	–	–
IV	6	–	–	–	3	–	–	–	9	–	4	–
High grade	–	–	–	–	–	–	–	–	–	–	1	–

Comparing the performance of the two tests, Spearman's correlation between the cumulative methylation of the two-marker set as determined by TAM-MSP and QM-MSP was highly similar (rho = 0.859 *P* < 2.2e−16) (Additional file 4: Fig. S4d).

### TAM-MSP analysis of methylation markers in blood

**Fig. 5:**
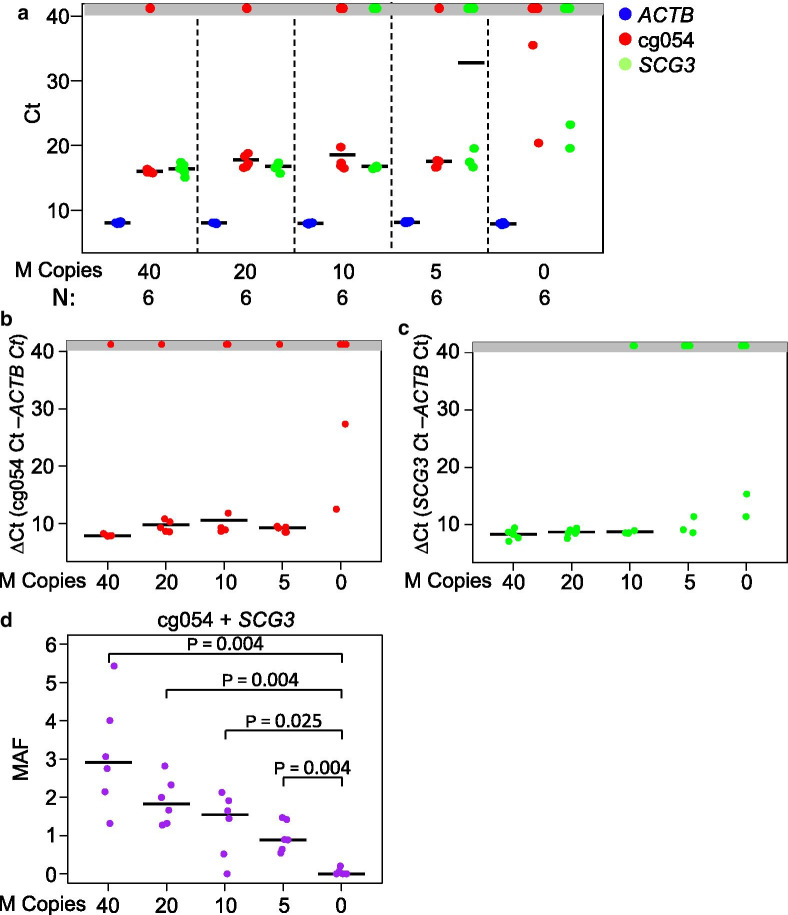
Determination of the limit of detection of methylated cfDNA spiked into plasma from healthy individuals. A serial dilution of Sss1-treated plasma cfDNA, ranging from 40 to 5 copies of methylated cfDNA, was spiked into healthy pooled plasma and analyzed with the TAM-MSP assay. Each dilution point was repeated with 6 replicates. **a** The Cts for *ACTB*, cg054 and *SCG3* for all dilution replicates showed a strong reproducibility of *ACTB* at all spike-in dilutions. **b** Marker cg054 shows no amplification in the healthy plasma (0 copies of spike in DNA) in 4 of 6 replicates and had high ∆Cts. **c**. Marker *SCG3* shows no amplification in the healthy plasma (0 copies of spike in DNA) in 4 of 6 replicates and had high ∆Cts. **d** The total MAF at all dilutions was significantly different from the healthy plasma without spiked-in of methylated cfDNA. The dots in the gray box represent a failure of the marker to cross the cycle threshold. *N* number of samples, *P* Mann–Whitney statistics, *MAF* methylation allele frequency

To investigate the methylation levels of cfDNA in healthy plasma and to determine the limit of detection of methylated DNA by the TAM-MSP assay, we spiked 0.5 ml of pooled healthy plasma with 40, 20, 10, 5 and 0 copies of fully methylated plasma cfDNA and measured the Ct values of all markers (Fig. 5a). Examined individually, cg054 failed to amplify in 1 of 6 spike-in replicates at 40 copies, 1/6 at 20 copies, 2/6 at 10 copies and 1/6 at 5 copies of methylated cfDNA (Fig. 5b). *SCG3* failed to amplify at 2 of the 6 spike-in replicates at 10 copies and 3/6 replicates at 5 copies (Fig. 5c). Only a single spike-in replicate at 10 copies failed to amplify both markers. The plasma replicates with no spiked copies (0) had no detectable methylation in 4/6 replicates. Importantly, the TAM-MSP assay was able to distinguish between 5 copies of spiked-in methylated DNA from healthy plasma cfDNA with statistical significance when expressed as methylation allele frequency (MAF) (Mann–Whitney *P* = 0.004) (Fig. 5d). This demonstration of very low to absent methylation when measured by TAM-MSP in the pooled plasma samples is in contrast to higher levels reported in array data in pooled plasma (Additional file 2: Fig. S2) and illustrates the difficulty in reproducibly detecting methylation at these very low levels.

We performed a small pilot testing the methylation markers by TAM-MSP in plasma from 15 PCNSL patients, 5 other CNS patients and 6 healthy individuals. Our results showed that 3 of the 15 (20%) PCNSL plasma samples had detectable levels of methylation, as measured by MAF (ranging from 1.97 to 89.69 MAF), at levels higher than any of the other CNS tumors and healthy individuals (ranging from 0.00 to 1.85 MAF) (Table 2). The markers cg054 and *SCG3* were detected in 8/15 (53%) and 4/15 (27%) PCNSL plasma samples, respectively. However, the cg054 was also detectable in 3/5 glioma/glioblastoma plasma samples albeit at lower levels (ranging from 0.14 to 1.18 MAF) and in 1/6 healthy samples (0.02 MAF). The *SCG3* marker was detected in one other CNS plasma (1.61 MAF) diagnosed with an infiltrating glioma tumor with a H3-K27M mutation which alters the epigenetic landscape of the tumor [25, 26] and 3/6 of the healthy plasma samples (ranging from 0.24 to 1.37 MAF).

**Table 2:** Detection of methylation markers, cg054 and *SCG3,* by TAM-MSP in plasma

ID	Diagnosis	cg054/*ACTB* copies	*SCG3*/*ACTB* copies	cg054 MAF (%)	*SCG3* MAF (%)	Total MAF (%)
PCNSL_1	PCNSL	1/660	0/660	0.20	0.00	0.20
PCNSL_2	PCNSL	8/1867	15/1867	0.45	0.80	1.25
PCNSL_3	PCNSL	84/2496	79/2496	3.37	3.15	6.52
PCNSL_4	PCNSL	9/2071	32/2071	0.42	1.55	1.97
PCNSL_5	PCNSL	1408/2826	1127/2826	49.82	39.88	89.69
PCNSL_6	PCNSL	0/2131	0/2131	0.00	0.00	0.00
PCNSL_7	PCNSL	0/1536	0/1536	0.00	0.00	0.00
PCNSL_8	PCNSL	0/243	0/243	0.00	0.00	0.00
PCNSL_9	PCNSL	54/5158	0/5158	1.04	0.00	1.04
PCNSL_10	PCNSL	21/2051	0/2051	1.03	0.00	1.03
PCNSL_11	PCNSL	0/76	0/76	0.00	0.00	0.00
PCNSL_12	PCNSL	0/7829	0/7829	0.00	0.00	0.00
PCNSL_13	PCNSL	0/1744	0/1744	0.00	0.00	0.00
PCNSL_14	PCNSL	2/1846	0/1846	0.09	0.00	0.09
PCNSL_15	PCNSL	0/37	0/37	0.00	0.00	0.00
Other_1	GBM	2/1279	0/1279	0.14	0.00	0.14
Other_2	IG^a^	6/2468	40/2468	0.24	1.61	1.85
Other_3	GBM	0/687	0/687	0.00	0.00	0.00
Other_4	GBM	13/115	0/1115	1.18	0.00	1.18
Other_5	DMG	0/1093	0/1093	0.00	0.00	0.00
Healthy_1	NA	0/1756	4/1756	0.02	0.24	0.26
Healthy_2	NA	0/2099	16/2099	0.00	0.76	0.76
Healthy_3	NA	0/2414	0/2414	0.00	0.00	0.00
Healthy_4	NA	0/1373	19/1373	0.00	1.37	1.37
Healthy_5	NA	0/1529	0/1529	0.00	0.00	0.00
Healthy_6	NA	0/1307	0/1307	0.00	0.00	0.00

*PCNSL* primary central nervous system lymphoma, *MAF* methylation allele frequency, other CNS tumors, *GBM* glioblastoma multiformae, *DMG* diffuse midline glioma, *IG* infiltrating glioma

^a^H3-K27M mutation

## Discussion

In this study, we have identified CpG sites that distinguish between PCNSL and other CNS tumors with great accuracy by in silico analysis of large publicly available TCGA and GEO’s 450K methylation array databases using a new Methylation Outlier program. Many of these markers were highly methylated (*β* value greater than 0.20) in multiple B-cell derived cancers including ALL and CLL but had significantly lower methylation levels in normal B-cells, B-cell precursors, and hematopoietic stem cells. Next, we tested the markers in a sample set of FFPE tissues of PCNSL and eight other types of CNS tumors using our newly developed laboratory assay, TAM-MSP. We showed that TAM-MSP assay performance was comparable to that of the well-established QM-MSP laboratory method. Furthermore, the TAM-MSP assay could distinguish as few as 5 copies of DNA spiked into 500 ul of plasma although there is high variance when measuring low copy numbers of methylated DNA that could give rise to errors in detection of cancer in liquid biopsies. In a small pilot study using PCNSL patient plasma samples, we successfully detected methylation markers in 3/15 samples at higher levels than those seen in plasma from normal individuals or patient with other CNS tumors. We concluded that the Methylation Outlier method used for identifying and verifying the markers performed efficiently whose findings were analytically validated in FFPE tissues with great accuracy. Whether these markers provide the sensitivity and specificity needed to detect PCNSL in cfDNA in liquid biopsies is yet to be determined.

Many studies have used high-density methylation arrays to identify markers which are then verified and used for qMSP assays. Through the reverse engineering of previously identified 450K methylation markers that have shown to have reproducible utility in different qMSP assays, we developed the heuristic rules for the Methylation Outlier Detector program [16, 27, 28]. Although these simple heuristic rules may not suitable to address all biological questions, we have shown here that the markers identified by this program could be developed successfully for at least two quantitative MSP assays, TAM-MSP and QM-MSP, and retained a level of accuracy equal to, or better than those observed in the methylation arrays. We also show the limitations of using array information to identify markers suitable for qMSP analysis of DNA methylation markers in plasma samples.

TAM-MSP includes specific considerations in its design that are especially pertinent to measuring cfDNA in liquid biopsies. Several studies have shown that the size of ctDNA in plasma is approximately 165 bp [29–31]. In TAM-MSP, by placing primers within the methylated region in the first multiplexed amplification step, we achieved two goals. First, we increased the efficiency of the PCR by amplifying just the regions of DNA that are informative for the assay, and second, the amplicon size was decreased to be within 150 bp, closer to the size of the DNA fragments found in circulation.

The two templates for a liquid biopsy of PCNSL patients are CSF and blood. Accessing peripheral blood requires a simple blood draw, whereas the collection of CSF requires a lumbar puncture or placement of an Ommaya reservoir, which is certainly more invasive. However, if a CSF-based, PCNSL-specific marker was identified, it could obviate the need of a tissue biopsy. A lumbar puncture, which is already part of standard workup for PCNSL, would possibly be preferable to an invasive biopsy.

Liquid biopsies generally require the measurement of small quantities of tumor DNA in the presence of large excess of shed normal DNA. In case the amount of shed DNA from PCNSL is below the limits of detection as shown by our findings using just 0.5 ml of plasma, CSF appears as the more promising source of cfDNA, as the tumor DNA in CSF is expected to be present at a higher level compared to normal than in peripheral blood. Furthermore, this route bypasses the blood–brain barrier which may limit the entry of ctDNA into peripheral circulation. Prior studies have illustrated that tumor-specific genetic alterations can be detected in CSF, but at lower frequency in plasma of patients with cancers of the CNS, including PCNSL [10, 32–35]. However, the methylation status for these markers in CSF is currently unknown.

## Conclusions

We have demonstrated the success of a new biomarker search program to identify promising, novel markers to distinguish between PCNSL and other common CNS tumors. Using quantitative multiplexed PCR assays, we have validated their utility in primary tumor tissues of this rare cancer. This is an important step to developing minimally invasive assays for this disease. Further studies are needed to accurately measure the sensitive and specificity of this new assay to detect the methylated marker panel in CSF of patients with PCNSL. This test, successfully implemented, could avoid invasive brain surgery and decrease lag time to definitive antineoplastic therapy.

## Methods

### Study design

In addition to FFPE samples selected from our archives and assayed in our laboratories, we used several public datasets.

The TCGA Dataset: For marker selection, data were downloaded from TCGA (HumanMethylation 450K) and consisted of the lymphoid neoplasm, diffuse large B-cell lymphoma (DLBCL, *N* = 48), glioblastoma and lower-grade gliomas (GBM and LGG, *N* = 656). These datasets were downloaded from the website firebrowse (http://firebrowse.org).

The GEO Dataset: For verification of the markers selected in the TCGA dataset, we used HumanMethylation 450K data downloaded from the Gene Expression Omnibus database (http://www.ncbi.nlm.nih.gov/geo/). This sample set consisted of: (1) PCNSL, all diffuse large B-cell lymphoma subtype, included in the GSE92676, database (*N* = 95) and (2) 11 non-PCNSL, primary brain tumors databases, GSE36278, GSE44684, GSE50774, GSE58218, GSE61044, GSE103659, GSE104210, GSE42882, GSE70787 and GSE85212 (*N* = 2112).

To test the performance of the two-marker panels, a second verification dataset from GEO (GSE90496) was downloaded. This dataset is comprised of 13 PCNSL and 2788 other tumors of the CNS and normal CNS tissues.

To determine whether the PCNSL markers identified in the TCGA dataset would be suitable for use in a plasma assay, publicly available datasets of methylome data (HumanMethylation 450K) of normal brain (GSE128601), whole blood (GSE40279), buffy coat (GSE109914), healthy pooled plasma cfDNA and cfDNA from plasma from healthy individuals (GSE122126) and were utilized.

To investigate whether the PCNSL markers are specific for B-cell cancers or for normal B-cells, hematopoietic stem cells (GSE63409), B-cell precursors (GSE45461), B-cell (GSE59250), acute lymphoblastic leukemia (ALL) (GSE67043) and chronic lymphocytic leukemia (CLL) (Bioconductor package (“BloodCancerMultiOmics2017”)) were analyzed.

The Wuhan Clinical Sample Set: This set consisted of archival FFPE tissues of PCNSL (*N* = 25), including GCB and non-GCB, and eight different types of CNS tumors (total *N *= 25: glioblastoma, *N* = 6; astrocytoma, *N* = 4; ependymoma, *N* = 2; germinoma, *N* = 2; medulloblastoma, *N* = 3; oligodendroglioma, *N* = 4; meningioma, *N* = 3; solitary fibrous tumor, *N* = 1), collected at the Renmin Hospital of Wuhan University. Slides from all 50 cases were evaluated and confirmed independently by two clinical pathologists.

DNA from these samples was used to test the performance of markers that were selected in the TCGA datasets and verified in the GEO datasets.

Plasma was collected from newly diagnosed PCNSL (*N* = 15) and glioma and glioblastoma patients (*N* = 5) at Johns Hopkins Hospital. The 6 healthy plasma samples and the pooled plasma sample were purchased from BioIVT (K_2_EDTA plasma, BioIVT, Hicksville, NY).

### Statistical considerations

With a sample size of 25 tissues in each group, and a sensitivity/specificity of 100%, the statistic was estimated to within 11% percentage points (95% lower confidence bound = 0 89%). Likewise, based on a simulation of ROC analysis, assuming a true AUC of 0.98, corresponding to one misclassified sample out of 50, we established 0.95 as the 95% lower confidence bound on the area under the curve of 1.00. The sample size for the plasma pilot study was selected by the availability of previously collected samples.

### Marker identification

Code availability: A newly self-developed marker discovery program named the Methylation Outlier Detector GitHub-MethylationOutlierDetector (https://github.com/bdowns4/MethylationOutlierDetector.git) was used to identify methylation markers that distinguish between DLBCL and CNS tumors in the TCGA dataset. DNA methylation levels for the CpGs in the publicly accessible HumanMethylation 450K data are reported as β values, which is defined as the ratio of the intensity of the methylated allele to the overall intensity. We removed markers from the analysis if more than 5% of the data in the TCGA or GEO datasets had missing *β* values. This program first selected all the markers that had a *β* value greater than 0.20 in the DLBCL and primary tumors of the CNS. The selected markers were then filtered out if more than 5% of the GBM and LGG samples had a *β* value greater than 0.15. Finally, the markers were sorted by their odds ratio. These heuristic rules have been made based on our previous experience in discovering methylated markers used for assaying circulating tumor DNA. In this study, we selected the markers that had β value greater than 0.20 in all (48/48) of the DLBCL tumors and had a *β* value less than 0.15 in all (656/656) of the GBM and LGG samples.

### Marker verification

To test the ability of the markers to distinguish PCNSL from other PCNS tumors, the area under the curve (AUC) and median beta value for each of the markers were calculated from the GEO dataset. To test the performance of two-marker panels, a single methylation value was derived by adding the *β* values of the two markers together. The performance, as measured by area under the receiver operating characteristic curve (ROC/AUC), was calculated for each of the 28 combinations of two-marker panels.

To determine the specificity of the selected markers, in the GEO dataset, we compared *β* values of tumor samples with the methylation *β* values in several subpopulations of normal peripheral blood cells and with normal brain tissue.

### Marker testing with quantitative methylation-specific PCR

Marker testing was performed using FFPE tissue (*N* = 50) from Wuhan Clinical Sample Set. DNA was extracted from 6-micron sections and treated with sodium bisulfite as previously described [27, 36, 37] prior to amplification by TAM-MSP (described below) or QM-MSP [23, 24]. For analysis of plasma, cell-free DNA (cfDNA) was extracted from 500 ul of plasma using the Quick-cfDNA serum and Plasma Kit (Zymo Research, D4076) following the manufacturer’s protocol and was eluted with 45 ul DNA elution buffer (Zymo Research, D3004-4), sodium bisulfite treated [27, 36, 37] and subjected to TAM-MSP analysis.

### Quantitative multiplexed-methylation-specific PCR (QM-MSP)

The QM-MSP assay was used to test the selected markers in bisulfite-treated DNA from FFPE tissues [23, 24]. QM-MSP data were expressed as Cumulative Methylation Index (CMI), the sum of percent methylation for each gene in the panel [23, 24]. The sequences of QM-MSP primers and probes (ThermoFisher Scientific) are listed in Additional file 6: Table S2. All probes for the methylated target were labeled with FAM, and the probes for the unmethylated targets were labeled with VIC. Both probes used TAMRA as quencher.

### Tailed amplicon multiplexed-methylation-specific PCR (TAM-MSP)

The TAM-MSP assay procedure consists of two sequential PCRs. In Step 1 PCR, (multiplexed generation of tailed end amplicons), 2–10 µl (of 100 ul lysate from a FFPE tissue section or cfDNA) of sodium bisulfite-treated DNA was added to 48 µl of reaction buffer [1.25 mM deoxynucleotide triphosphates, 16.6 mM (NH4)2SO4, 67 mM Tris (pH 8.8), 6.7 mM MgCl2, 10 mM β-mercaptoethanol, 0.1% DMSO, 5 unit of Platinum Taq (Invitrogen) and 400 nM each of the forward and reverse primers]. Conditions used for this PCR step were: 95 °C for 5 min, followed by 35 cycles of 95 °C for 30 s, 56 °C for 45 s and 72 °C for 45 s, with a final extension cycle of 72 °C for 7 min. The PCR products were diluted to 200 µl with reaction buffer and stored at − 20 °C.

For Step 2 PCR (quantitative multiplexed PCR of amplicons), 1 µl of the 1:5 diluted PCR product from Step 1 was further diluted 1:100. 1 ul of the diluted DNA was added to 20 µl qMSP reaction buffer [16.6 mM (NH4)2SO4, 67.0 mM Tris (pH 8.8), 6.7 mM MgCl2, 10.0 mM β-mercaptoethanol, 0.1% DMSO, 200 µM deoxynucleotide triphosphates, 1.25 units Ramp Taq (Thomas Scientific), 50 ug/ml tRNA (Invitrogen) and 300 nM ROX (Invitrogen)], 700 nM each of primers (forward and reverse) and 200 nM labeled probe (Applied Biosystems). The reaction was carried out in a 96-well reaction plate in a 7500 Fast Real-Time PCR (Applied Biosystems). The reaction conditions were: 95 °C for 7 min, followed by 40 cycles of 95 °C for 15 s and 62 °C for 1 min.

To quantitatively assess three markers in a single reaction, *ACTB*, marker 1 and marker 2 probes were labeled with fluorophores FAM, VIC and NED, respectively, while the fluorophore MGB-NFQ was used as the common quencher for all three probes. The sequences of the TAM-MSP primers and probes are listed in Additional file 7: Table S3.

For calculating the copies of *ACTB* DNA and % methylation, reported as cumulative methylation index (CMI) and/ or methylation allele frequency (MAF), the ∆∆Ct method {100/(2^-[(methylated control Ct Marker- methylated control Ct *ACTB*) − (Sample Ct Marker – sample Ct *ACTB*)]} was used. The methylated control DNA used was a fully methylated human DNA control (Promega, N1231). The copy number of *ACTB* and methylated DNA was calculated by measuring the difference of Ct values between the *ACTB* of the fully methylated human control DNA at 2 ng (606 copies) and the ACTB Ct signal of the plasma sample.

Inter-assay reproducibility for TAM-MSP analysis was calculated from the standard curves of mixtures of fully methylated DNA [SssI (New England Biolabs, M0226S)-treated human sperm DNA (HSD)], and unmethylated DNA (untreated HSD), in mixtures yielding 100–3% methylation in six replicates.

Inter-platform reproducibility was tested by comparing the cumulative methylation of the two-marker panel obtained by the TAM-MSP and QM-MSP assays.

To determine the limit of detection of methylated markers in ctDNA, TAM-MSP analysis was performed by spiking in 40, 20, 10, 5 and 0 copies of Sss1-treated cfDNA from healthy plasma into 0.5 ml of pooled normal plasma (human pooled plasma, K_2_EDTA anticoagulated or pooled serum, BioIVT, Hicksville, NY). Prior to spiking, the Sss1-treated plasma cfDNA was concentrated by the Zymo DNA Clean and Concentrator-5 kit (Zymo Research, D4013) and was quantified by nanodrop. Further, we confirmed copy numbers in the spike-in by comparing the Ct values for each gene detected in the spiked samples to known amounts of commercial grade fully methylated human DNA control (Promega, N1231). Each reaction was repeated six times.

Statistical analysis: The R software (version 3.6.0) was used. A custom script and the R package pROC were used to construct the receiver operator characteristic (ROC) plot and to calculate the area under the curve (AUC) and derive 95% confidence interval (CI) for each of the markers and for each of the 28 combinations of two-marker panels, using cumulative β values, in the GEO Dataset [38]. The R package pheatmap was used to generate the heatmap plots [39]. The boxplots were made with the R function boxplot. Mann–Whitney *P* values were calculated with the R function wilcox.test. Histogram plots were made using GraphPad Prism version 8.1.2. Interassay reproducibility was reported as coefficient of variation (CV) as calculated by GraphPad Prism version 8.1.2. Inter-platform reproducibility was tested following analysis of the Wuhan Clinical Sample Set by both TAM-MSP and QM-MSP assays. The R function cor.test was used to calculate the Spearman rho value and *P* value.

## Supplementary Information

**Additional file 1: Fig. S1**. Marker methylation in PCNSL compared to normal peripheral blood cell subgroups and other B-cell cancers. Box plots show the β-values for methylation as assessed in GEO datasets of PCNSL tumors compared to subpopulations of normal peripheral blood cells and B-cell derived cancers. Exceapt for marker NCOR2, the B-cell derived cancers show higher methylation β-values for these genes than hematopoietic stem cells, B-cell precursors, and B-cells. N = number of samples; Mann Whitney: ** = P < 0.001, * = P < 0.05, N.S. non-significant.

**Additional file 2: Fig. S2**. Marker methylation in PCNSL normal brain tissues, whole blood, buffy coat and plasma. Box plots show the methylation status, β-values, as assessed from GEO datasets of PCNSL tumors compared to subpopulations of normal blood samples and normal brain tissue. Marker methylation β-values in PCNSL, in each of the 8 markers identified in the TCGA datasets, is significantly higher than each of the subpopulations of whole blood, buffy coat, plasma and normal brain tissue. N = number of samples; ** = P < 0.001, * = P < 0.05, N.S. non-significant Mann-Whitney.

**Additional file 3: Fig. S3.** Performance characteristics of TAM-MSP.** a** A standard curve was generated by mixing fully methylated (Sss1-treated) human sperm DNA (HSD) with fully unmethylated HSD to yield dilutions of 3-100 % methylation. Each dot represents the average ∆Ct value (Ct of sample-Ct of ACTB) of 6 replicates.** b** Inter-assay reproducibility was calculated from the ∆Cts generated from the standard curve in** a**. M = methylated; N = number of replicates; CV = coefficient of variation; P = Mann-Whitney statistics.

**Additional file 4: Fig. S4**. Testing of the 5 markers independently, and as a two-marker panel, in the tissue sample set.** a** Using QM-MSP, the 5 markers, (cg054, SCG3, DOCK1, GRIK1, and KCNH7) analyzed independently, and as a two-marker panel, cg054 and SCG3, could distinguish PCNSL (N = 25) from 8 other CNS tumors (N = 25) with a high level of accuracy.** b** The histogram displays the contribution (percent methylation) of each of markers of the panel to detect PCNSL.** c** Analysis of receiver operator characteristics (ROC, inset) show that the two-marker panel performed with a high level of accuracy with AUC of 1.00 (CI: 0.95-1.00).** d** Comparison of performance of TAM-MSP and QM-MSP. Dot plot shows the cumulative methylation values of the two-marker panel as determined by TAM-MSP and QM-MSP. Methylation detection by the two methods show a high degree of correlation, Spearman correlation, rho=0.859.** = Mann-Whitney P < 0.001; CMI = cumulative methylation index; N = number of samples; P = Mann-Whitney statistics; AUC = area under the curve.

**Additional file 5: Table S1**. Marker characteristics.

**Additional file 6: Table S2**. QM-MSP primer and probe characteristics.

**Additional file 7: Table S3**. TAM-MSP primer and probe characteristics.

## Data Availability

All datasets are available to be downloaded from Geo, TCGA and Bioconductor and are described in the manuscript.

## Abbreviations

PCNSL: Primary central nervous system lymphoma
CNS: Central nervous system
TCGA: The Cancer Genome Atlas
DLBCL: Diffuse large B-cell lymphoma
ROC: Receiver operating characteristics
AUC: Area under the curve
GEO: Gene Expression Omnibus
TAM-MSP: Tailed Amplicon Multiplexed-Methylation-Specific PCR
CI: Confidence interval
ABC: Activated B-cell
GCB: Germinal center B-cell
PCNS: Primary central nervous system
CSF: Cerebrospinal fluid
450K: Infinium HumanMethylation 450K BeadChip
FFPE: Formalin fixed, paraffin embedded
ctDNA: Circulating tumor DNA
GMB: Glioblastoma
LGG: Lower-grade gliomas
ALL: Acute lymphoblastic leukemia
CLL: Chronic lymphocytic leukemia
cfDNA: Circulating cell-free DNA
QM-MSP: Quantitative multiplexed-methylation-specific PCR
CMI: Cumulative Methylation Index
MAF: Methylation allele frequency
HSD: Human sperm DNA
CV: Coefficient of variation
