# Supplementary material for: Methylated markers accurately distinguish primary central nervous system lymphomas (PCNSL) from other CNS tumors
Source: Clin Epigenetics. 2021 May 5;13:104. doi: 10.1186/s13148-021-01091-9 (PMC8097855; doi:10.1186/s13148-021-01091-9)
Supplement: Supplementary file 1 — Additional file 1: Fig. S1. Marker methylation in PCNSL compared to normal peripheral blood cell subgroups and other B-cell cancers. Box plots show the β-values for methylation as assessed in GEO datasets of PCNSL tumors compared to subpopulations of normal peripheral blood cells and B-cell derived cancers. Exceapt for marker NCOR2, the B-cell derived cancers show higher methylation β-values for these genes than hematopoietic stem cells, B-cell precursors, and B-cells. N = number of samples; Mann Whitney: ** = P < 0.001, * = P < 0.05, N.S. non-significant. [file 13148_2021_1091_MOESM1_ESM.pptx]

## Slide 1
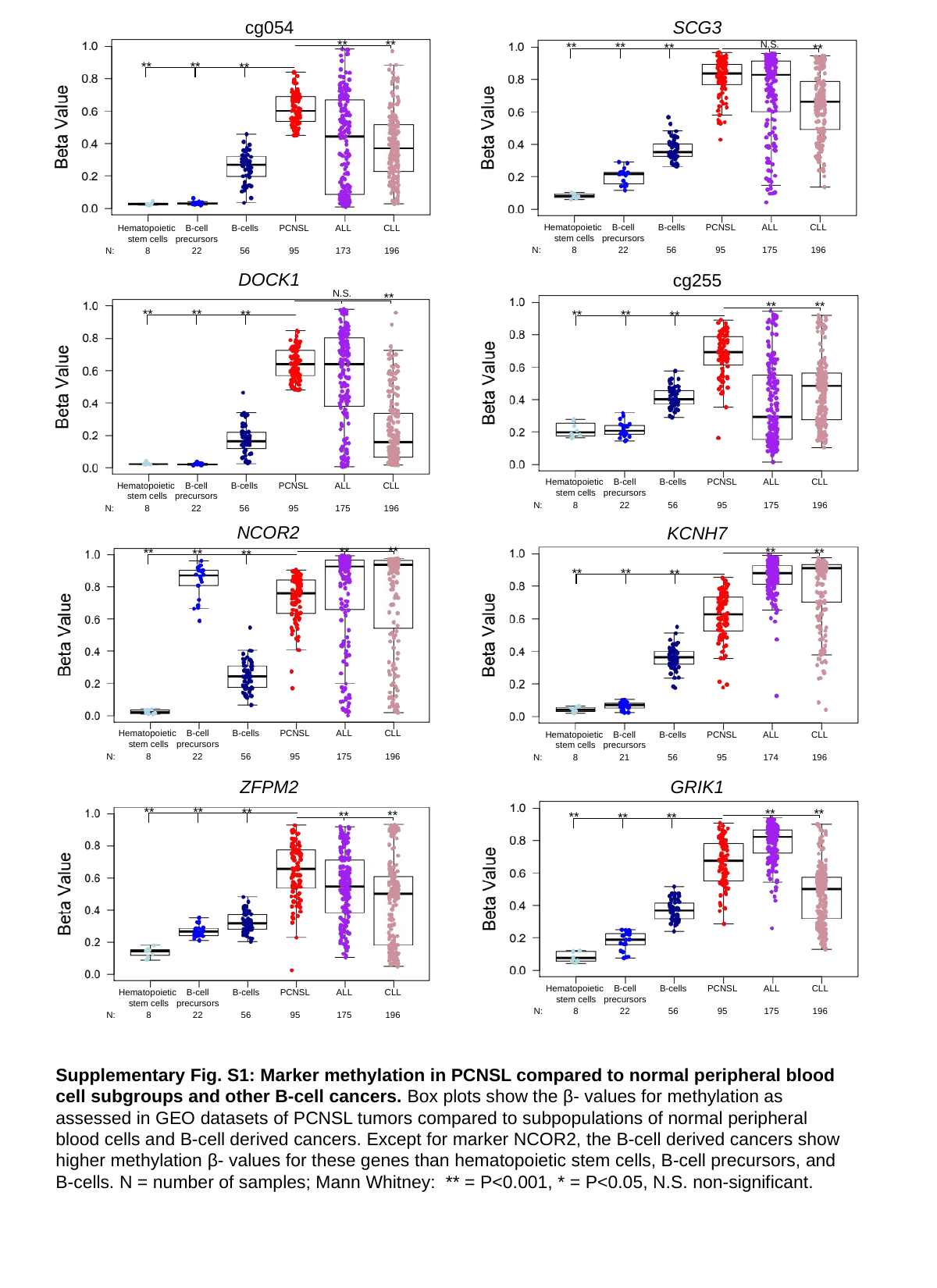

cg054
SCG3
**
**
N.S.
**
**
**
**
**
**
**
Hematopoietic
stem cells
B-cell
precursors
B-cells
PCNSL
ALL
CLL
N:
8
22
56
95
175
196
Hematopoietic
stem cells
B-cell
precursors
B-cells
PCNSL
ALL
CLL
N:
8
22
56
95
173
196
DOCK1
cg255
N.S.
**
**
**
**
**
**
**
**
**
Hematopoietic
stem cells
B-cell
precursors
B-cells
PCNSL
ALL
CLL
N:
8
22
56
95
175
196
Hematopoietic
stem cells
B-cell
precursors
B-cells
PCNSL
ALL
CLL
N:
8
22
56
95
175
196
NCOR2
KCNH7
**
**
**
**
**
**
**
**
**
**
Hematopoietic
stem cells
B-cell
precursors
B-cells
PCNSL
ALL
CLL
N:
8
22
56
95
175
196
Hematopoietic
stem cells
B-cell
precursors
B-cells
PCNSL
ALL
CLL
N:
8
21
56
95
174
196
ZFPM2
GRIK1
**
**
**
**
**
**
**
**
**
**
Hematopoietic
stem cells
B-cell
precursors
B-cells
PCNSL
ALL
CLL
N:
8
22
56
95
175
196
Hematopoietic
stem cells
B-cell
precursors
B-cells
PCNSL
ALL
CLL
N:
8
22
56
95
175
196
Supplementary Fig. S1: Marker methylation in PCNSL compared to normal peripheral blood cell subgroups and other B-cell cancers. Box plots show the β- values for methylation as assessed in GEO datasets of PCNSL tumors compared to subpopulations of normal peripheral blood cells and B-cell derived cancers. Except for marker NCOR2, the B-cell derived cancers show higher methylation β- values for these genes than hematopoietic stem cells, B-cell precursors, and B-cells. N = number of samples; Mann Whitney: ** = P<0.001, * = P<0.05, N.S. non-significant.
