# Supplementary material for: Methylated markers accurately distinguish primary central nervous system lymphomas (PCNSL) from other CNS tumors
Source: Clin Epigenetics. 2021 May 5;13:104. doi: 10.1186/s13148-021-01091-9 (PMC8097855; doi:10.1186/s13148-021-01091-9)
Supplement: Supplementary file 2 — Additional file 2: Fig. S2. Marker methylation in PCNSL normal brain tissues, whole blood, buffy coat and plasma. Box plots show the methylation status, β-values, as assessed from GEO datasets of PCNSL tumors compared to subpopulations of normal blood samples and normal brain tissue. Marker methylation β-values in PCNSL, in each of the 8 markers identified in the TCGA datasets, is significantly higher than each of the subpopulations of whole blood, buffy coat, plasma and normal brain tissue. N = number of samples; ** = P < 0.001, * = P < 0.05, N.S. non-significant Mann-Whitney. [file 13148_2021_1091_MOESM2_ESM.pptx]

## Slide 1
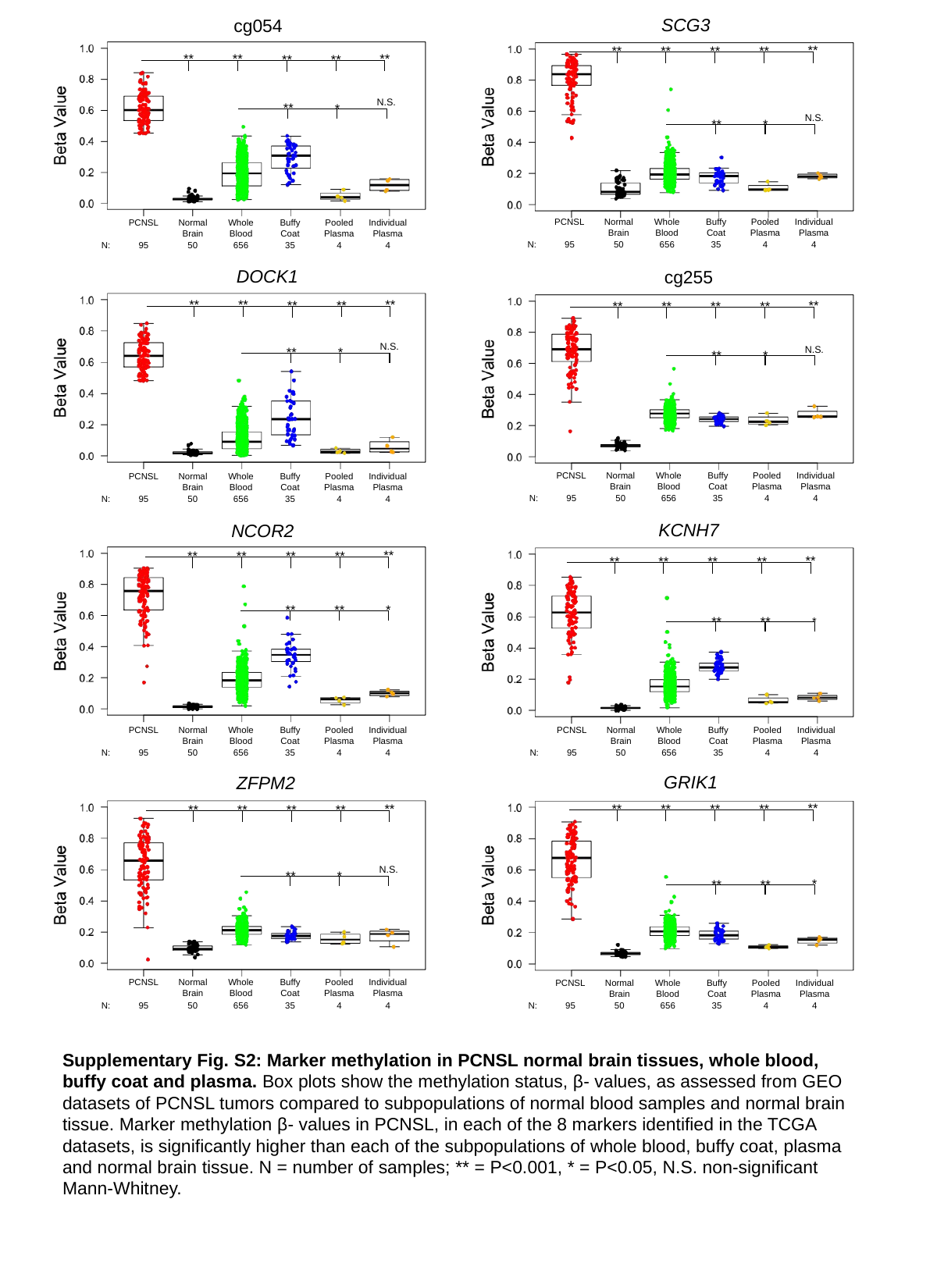

cg054
PCNSL
Normal
Brain
Whole
Blood
Buffy
Coat
Pooled
Plasma
Individual
Plasma
N:
95
50
656
35
4
4
SCG3
PCNSL
Normal
Brain
Whole
Blood
Buffy
Coat
Pooled
Plasma
Individual
Plasma
N:
95
50
656
35
4
4
**
**
**
**
**
**
**
**
**
**
N.S.
**
*
N.S.
**
*
DOCK1
PCNSL
Normal
Brain
Whole
Blood
Buffy
Coat
Pooled
Plasma
Individual
Plasma
N:
95
50
656
35
4
4
cg255
PCNSL
Normal
Brain
Whole
Blood
Buffy
Coat
Pooled
Plasma
Individual
Plasma
N:
95
50
656
35
4
4
**
**
**
**
**
**
**
**
**
**
N.S.
**
*
N.S.
**
*
NCOR2
PCNSL
Normal
Brain
Whole
Blood
Buffy
Coat
Pooled
Plasma
Individual
Plasma
N:
95
50
656
35
4
4
KCNH7
PCNSL
Normal
Brain
Whole
Blood
Buffy
Coat
Pooled
Plasma
Individual
Plasma
N:
95
50
656
35
4
4
**
**
**
**
**
**
**
**
**
**
*
**
**
**
**
*
GRIK1
PCNSL
Normal
Brain
Whole
Blood
Buffy
Coat
Pooled
Plasma
Individual
Plasma
N:
95
50
656
35
4
4
ZFPM2
PCNSL
Normal
Brain
Whole
Blood
Buffy
Coat
Pooled
Plasma
Individual
Plasma
N:
95
50
656
35
4
4
**
**
**
**
**
**
**
**
**
**
N.S.
**
*
*
**
**
Supplementary Fig. S2: Marker methylation in PCNSL normal brain tissues, whole blood, buffy coat and plasma. Box plots show the methylation status, β- values, as assessed from GEO datasets of PCNSL tumors compared to subpopulations of normal blood samples and normal brain tissue. Marker methylation β- values in PCNSL, in each of the 8 markers identified in the TCGA datasets, is significantly higher than each of the subpopulations of whole blood, buffy coat, plasma and normal brain tissue. N = number of samples; ** = P<0.001, * = P<0.05, N.S. non-significant Mann-Whitney.
