# Supplementary material for: Methylated markers accurately distinguish primary central nervous system lymphomas (PCNSL) from other CNS tumors
Source: Clin Epigenetics. 2021 May 5;13:104. doi: 10.1186/s13148-021-01091-9 (PMC8097855; doi:10.1186/s13148-021-01091-9)
Supplement: Supplementary file 3 — Additional file 3: Fig. S3. Performance characteristics of TAM-MSP. a A standard curve was generated by mixing fully methylated (Sss1-treated) human sperm DNA (HSD) with fully unmethylated HSD to yield dilutions of 3-100 % methylation. Each dot represents the average ∆Ct value (Ct of sample-Ct of ACTB) of 6 replicates. b Inter-assay reproducibility was calculated from the ∆Cts generated from the standard curve in a. M = methylated; N = number of replicates; CV = coefficient of variation; P = Mann-Whitney statistics. [file 13148_2021_1091_MOESM3_ESM.pptx]

## Slide 1
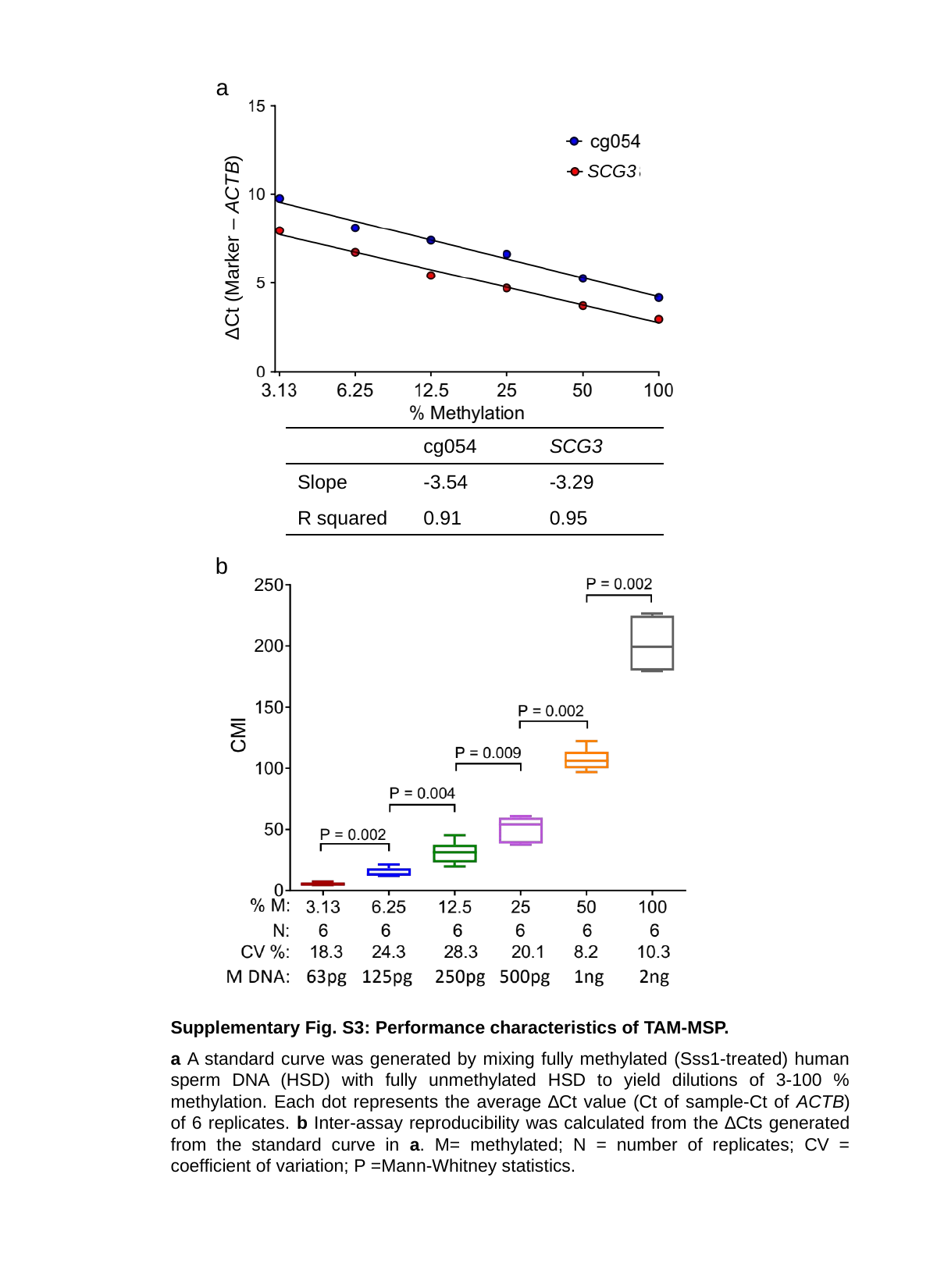

a
SCG3
∆Ct (Marker – ACTB)
| | cg054 | SCG3 |
| --- | --- | --- |
| Slope | -3.54 | -3.29 |
| R squared | 0.91 | 0.95 |
b
Supplementary Fig. S3: Performance characteristics of TAM-MSP.
a A standard curve was generated by mixing fully methylated (Sss1-treated) human sperm DNA (HSD) with fully unmethylated HSD to yield dilutions of 3-100 % methylation. Each dot represents the average ∆Ct value (Ct of sample-Ct of ACTB) of 6 replicates. b Inter-assay reproducibility was calculated from the ∆Cts generated from the standard curve in a. M= methylated; N = number of replicates; CV = coefficient of variation; P =Mann-Whitney statistics.
