# Supplementary material for: Methylated markers accurately distinguish primary central nervous system lymphomas (PCNSL) from other CNS tumors
Source: Clin Epigenetics. 2021 May 5;13:104. doi: 10.1186/s13148-021-01091-9 (PMC8097855; doi:10.1186/s13148-021-01091-9)
Supplement: Supplementary file 4 — Additional file 4: Fig. S4. Testing of the 5 markers independently, and as a two-marker panel, in the tissue sample set. a Using QM-MSP, the 5 markers, (cg054, SCG3, DOCK1, GRIK1, and KCNH7) analyzed independently, and as a two-marker panel, cg054 and SCG3, could distinguish PCNSL (N = 25) from 8 other CNS tumors (N = 25) with a high level of accuracy. b The histogram displays the contribution (percent methylation) of each of markers of the panel to detect PCNSL. c Analysis of receiver operator characteristics (ROC, inset) show that the two-marker panel performed with a high level of accuracy with AUC of 1.00 (CI: 0.95-1.00). d Comparison of performance of TAM-MSP and QM-MSP. Dot plot shows the cumulative methylation values of the two-marker panel as determined by TAM-MSP and QM-MSP. Methylation detection by the two methods show a high degree of correlation, Spearman correlation, rho=0.859.** = Mann-Whitney P < 0.001; CMI = cumulative methylation index; N = number of samples; P = Mann-Whitney statistics; AUC = area under the curve. [file 13148_2021_1091_MOESM4_ESM.pptx]

## Slide 1
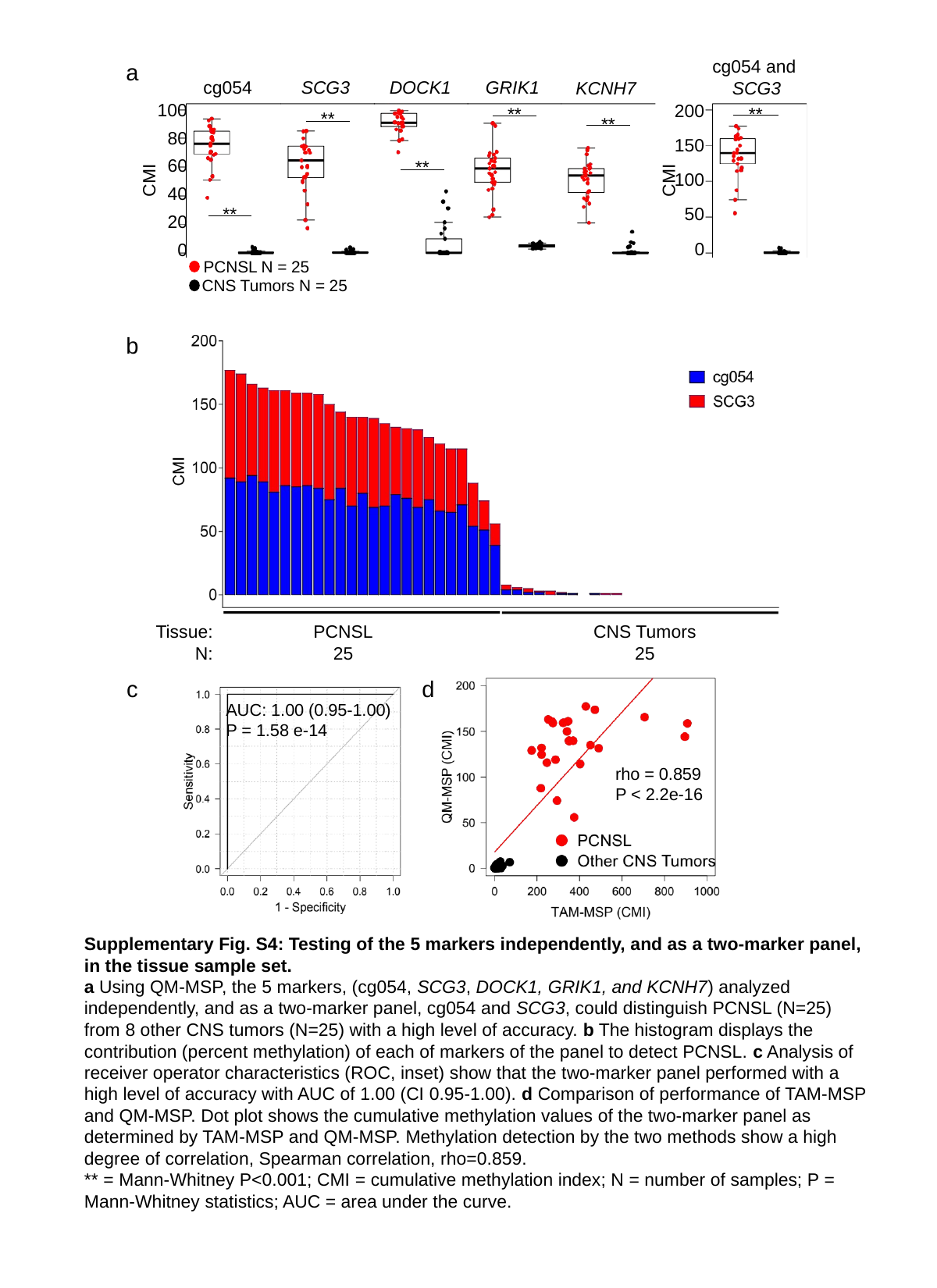

cg054 and
SCG3
a
cg054
SCG3
DOCK1
GRIK1
KCNH7
100
80
60
40
20
0
200
150
100
50
0
**
**
**
**
**
CMI
CMI
**
PCNSL N = 25
CNS Tumors N = 25
b
Tissue:
N:
PCNSL
25
CNS Tumors
25
c
d
AUC: 1.00 (0.95-1.00)
P = 1.58 e-14
rho = 0.859
P < 2.2e-16
Supplementary Fig. S4: Testing of the 5 markers independently, and as a two-marker panel, in the tissue sample set.
a Using QM-MSP, the 5 markers, (cg054, SCG3, DOCK1, GRIK1, and KCNH7) analyzed independently, and as a two-marker panel, cg054 and SCG3, could distinguish PCNSL (N=25) from 8 other CNS tumors (N=25) with a high level of accuracy. b The histogram displays the contribution (percent methylation) of each of markers of the panel to detect PCNSL. c Analysis of receiver operator characteristics (ROC, inset) show that the two-marker panel performed with a high level of accuracy with AUC of 1.00 (CI 0.95-1.00). d Comparison of performance of TAM-MSP and QM-MSP. Dot plot shows the cumulative methylation values of the two-marker panel as determined by TAM-MSP and QM-MSP. Methylation detection by the two methods show a high degree of correlation, Spearman correlation, rho=0.859.
** = Mann-Whitney P<0.001; CMI = cumulative methylation index; N = number of samples; P = Mann-Whitney statistics; AUC = area under the curve.
