# Supplementary material for: Methylated markers accurately distinguish primary central nervous system lymphomas (PCNSL) from other CNS tumors
Source: Clin Epigenetics. 2021 May 5;13:104. doi: 10.1186/s13148-021-01091-9 (PMC8097855; doi:10.1186/s13148-021-01091-9)
Supplement: Supplementary file 5 — Additional file 5: Table S1. Marker characteristics. [file 13148_2021_1091_MOESM5_ESM.pptx]

## Slide 1
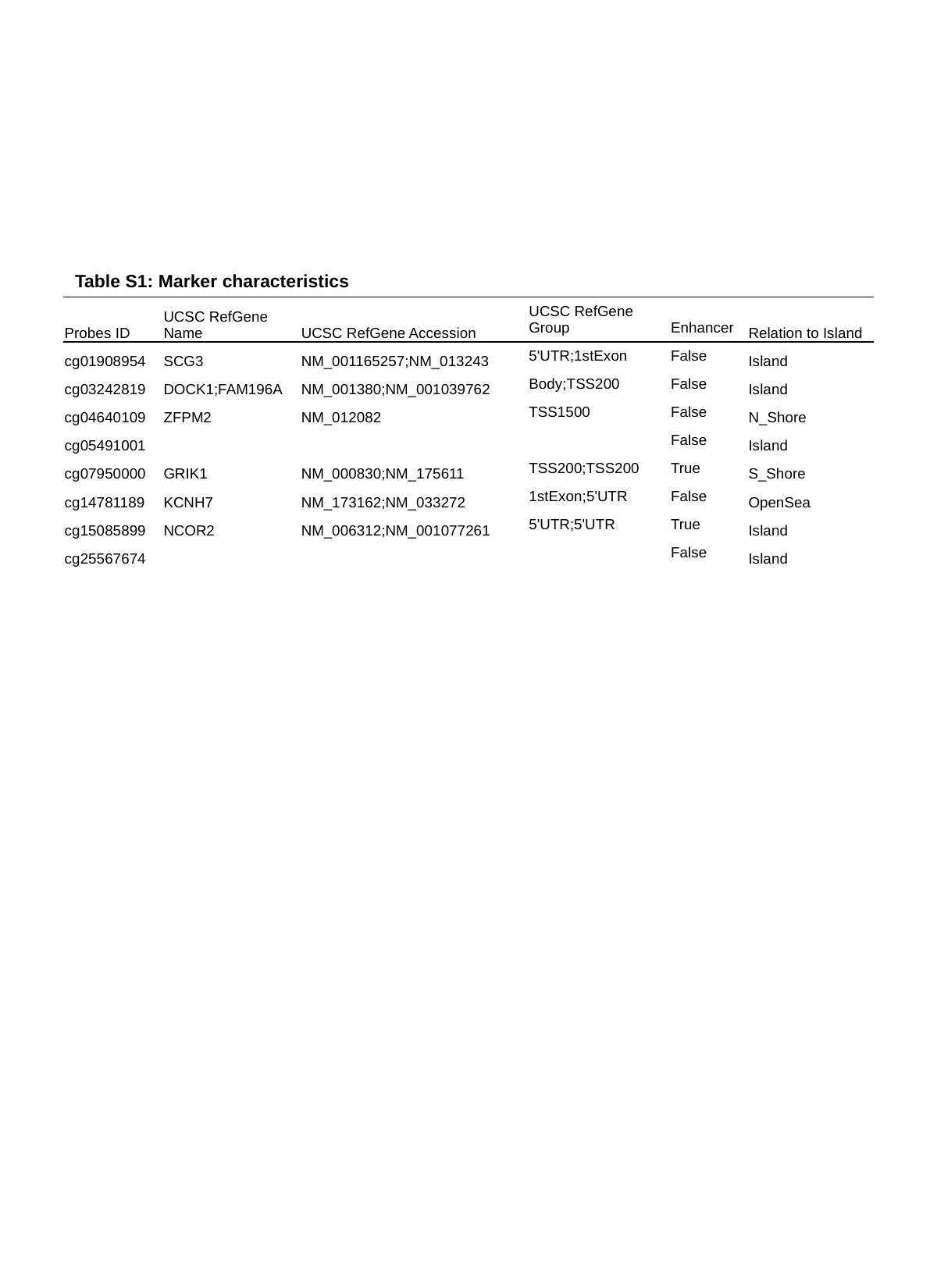

| Table S1: Marker characteristics | | | | | |
| --- | --- | --- | --- | --- | --- |
| Probes ID | UCSC RefGene Name | UCSC RefGene Accession | UCSC RefGene Group | Enhancer | Relation to Island |
| cg01908954 | SCG3 | NM\_001165257;NM\_013243 | 5'UTR;1stExon | False | Island |
| cg03242819 | DOCK1;FAM196A | NM\_001380;NM\_001039762 | Body;TSS200 | False | Island |
| cg04640109 | ZFPM2 | NM\_012082 | TSS1500 | False | N\_Shore |
| cg05491001 | | | | False | Island |
| cg07950000 | GRIK1 | NM\_000830;NM\_175611 | TSS200;TSS200 | True | S\_Shore |
| cg14781189 | KCNH7 | NM\_173162;NM\_033272 | 1stExon;5'UTR | False | OpenSea |
| cg15085899 | NCOR2 | NM\_006312;NM\_001077261 | 5'UTR;5'UTR | True | Island |
| cg25567674 | | | | False | Island |
