# Supplementary material for: Methylated markers accurately distinguish primary central nervous system lymphomas (PCNSL) from other CNS tumors
Source: Clin Epigenetics. 2021 May 5;13:104. doi: 10.1186/s13148-021-01091-9 (PMC8097855; doi:10.1186/s13148-021-01091-9)
Supplement: Supplementary file 6 — Additional file 6: Table S2. QM-MSP primer and probe characteristics. [file 13148_2021_1091_MOESM6_ESM.pptx]

## Slide 1
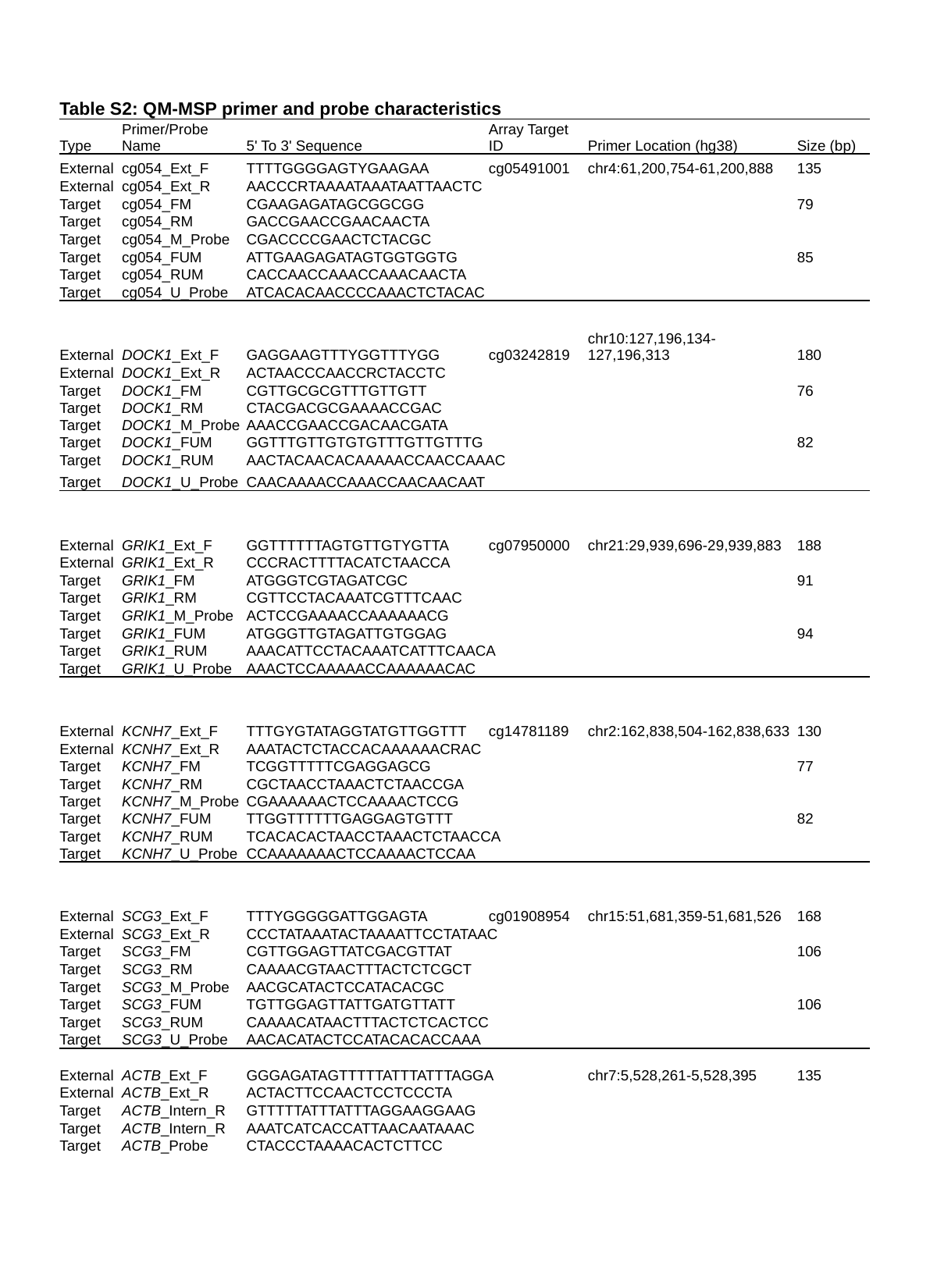

| Table S2: QM-MSP primer and probe characteristics | | | | | | |
| --- | --- | --- | --- | --- | --- | --- |
| Type | Primer/Probe Name | 5' To 3' Sequence | Array Target ID | Array Target ID | Primer Location (hg38) | Size (bp) |
| External | cg054\_Ext\_F | TTTTGGGGAGTYGAAGAA | cg05491001 | chr4:61,200,261-61,201,382 | chr4:61,200,754-61,200,888 | 135 |
| External | cg054\_Ext\_R | AACCCRTAAAATAAATAATTAACTC | | | | |
| Target | cg054\_FM | CGAAGAGATAGCGGCGG | | | | 79 |
| Target | cg054\_RM | GACCGAACCGAACAACTA | | | | |
| Target | cg054\_M\_Probe | CGACCCCGAACTCTACGC | | | | |
| Target | cg054\_FUM | ATTGAAGAGATAGTGGTGGTG | | | | 85 |
| Target | cg054\_RUM | CACCAACCAAACCAAACAACTA | | | | |
| Target | cg054\_U\_Probe | ATCACACAACCCCAAACTCTACAC | | | | |
| | | | | | | |
| External | DOCK1\_Ext\_F | GAGGAAGTTTYGGTTTYGG | cg03242819 | cg03242819 | chr10:127,196,134-127,196,313 | 180 |
| External | DOCK1\_Ext\_R | ACTAACCCAACCRCTACCTC | | | | |
| Target | DOCK1\_FM | CGTTGCGCGTTTGTTGTT | | | | 76 |
| Target | DOCK1\_RM | CTACGACGCGAAAACCGAC | | | | |
| Target | DOCK1\_M\_Probe | AAACCGAACCGACAACGATA | | | | |
| Target | DOCK1\_FUM | GGTTTGTTGTGTGTTTGTTGTTTG | | | | 82 |
| Target | DOCK1\_RUM | AACTACAACACAAAAACCAACCAAAC | | | | |
| Target | DOCK1\_U\_Probe | CAACAAAACCAAACCAACAACAAT | | | | |
| | | | | | | |
| External | GRIK1\_Ext\_F | GGTTTTTTAGTGTTGTYGTTA | cg07950000 | cg07950000 | chr21:29,939,696-29,939,883 | 188 |
| External | GRIK1\_Ext\_R | CCCRACTTTTACATCTAACCA | | | | |
| Target | GRIK1\_FM | ATGGGTCGTAGATCGC | | | | 91 |
| Target | GRIK1\_RM | CGTTCCTACAAATCGTTTCAAC | | | | |
| Target | GRIK1\_M\_Probe | ACTCCGAAAACCAAAAAACG | | | | |
| Target | GRIK1\_FUM | ATGGGTTGTAGATTGTGGAG | | | | 94 |
| Target | GRIK1\_RUM | AAACATTCCTACAAATCATTTCAACA | | | | |
| Target | GRIK1\_U\_Probe | AAACTCCAAAAACCAAAAAACAC | | | | |
| | | | | | | |
| External | KCNH7\_Ext\_F | TTTGYGTATAGGTATGTTGGTTT | cg14781189 | chr2:162,838,041-162,839,162 | chr2:162,838,504-162,838,633 | 130 |
| External | KCNH7\_Ext\_R | AAATACTCTACCACAAAAAACRAC | | | | |
| Target | KCNH7\_FM | TCGGTTTTTCGAGGAGCG | | | | 77 |
| Target | KCNH7\_RM | CGCTAACCTAAACTCTAACCGA | | | | |
| Target | KCNH7\_M\_Probe | CGAAAAAACTCCAAAACTCCG | | | | |
| Target | KCNH7\_FUM | TTGGTTTTTTGAGGAGTGTTT | | | | 82 |
| Target | KCNH7\_RUM | TCACACACTAACCTAAACTCTAACCA | | | | |
| Target | KCNH7\_U\_Probe | CCAAAAAAACTCCAAAACTCCAA | | | | |
| | | | | | | |
| External | SCG3\_Ext\_F | TTTYGGGGGATTGGAGTA | cg01908954 | cg01908954 | chr15:51,681,359-51,681,526 | 168 |
| External | SCG3\_Ext\_R | CCCTATAAATACTAAAATTCCTATAAC | | | | |
| Target | SCG3\_FM | CGTTGGAGTTATCGACGTTAT | | | | 106 |
| Target | SCG3\_RM | CAAAACGTAACTTTACTCTCGCT | | | | |
| Target | SCG3\_M\_Probe | AACGCATACTCCATACACGC | | | | |
| Target | SCG3\_FUM | TGTTGGAGTTATTGATGTTATT | | | | 106 |
| Target | SCG3\_RUM | CAAAACATAACTTTACTCTCACTCC | | | | |
| Target | SCG3\_U\_Probe | AACACATACTCCATACACACCAAA | | | | |
| | | | | | | |
| External | ACTB\_Ext\_F | GGGAGATAGTTTTTATTTATTTAGGA | | | chr7:5,528,261-5,528,395 | 135 |
| External | ACTB\_Ext\_R | ACTACTTCCAACTCCTCCCTA | | | | |
| Target | ACTB\_Intern\_R | GTTTTTATTTATTTAGGAAGGAAG | | | | |
| Target | ACTB\_Intern\_R | AAATCATCACCATTAACAATAAAC | | | | |
| Target | ACTB\_Probe | CTACCCTAAAACACTCTTCC | | | | |
