# Supplementary material for: Methylated markers accurately distinguish primary central nervous system lymphomas (PCNSL) from other CNS tumors
Source: Clin Epigenetics. 2021 May 5;13:104. doi: 10.1186/s13148-021-01091-9 (PMC8097855; doi:10.1186/s13148-021-01091-9)
Supplement: Supplementary file 7 — Additional file 7: Table S3. TAM-MSP primer and probe characteristics. [file 13148_2021_1091_MOESM7_ESM.pptx]

## Slide 1
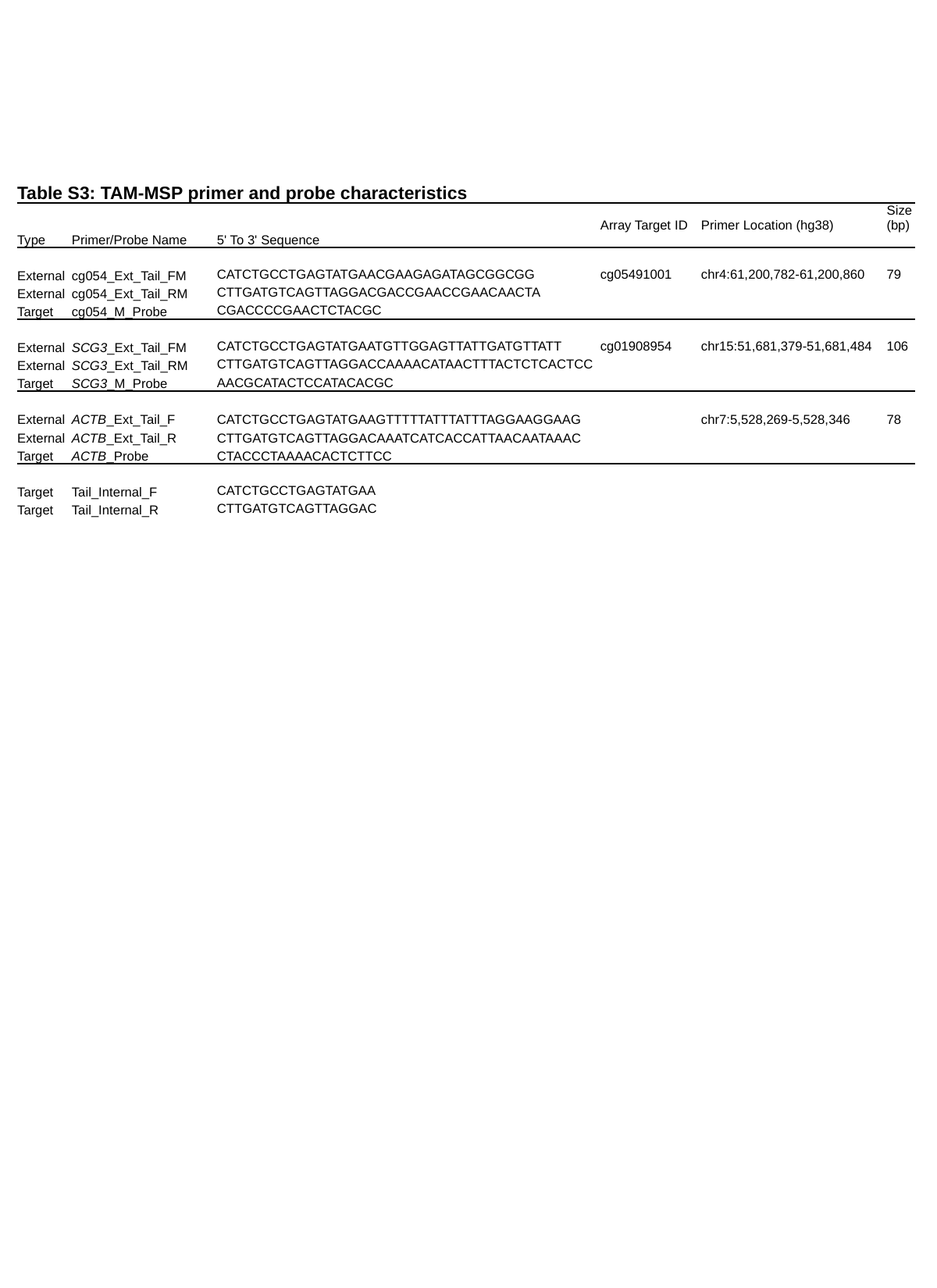

| Table S3: TAM-MSP primer and probe characteristics | | | | | |
| --- | --- | --- | --- | --- | --- |
| Type | Primer/Probe Name | 5' To 3' Sequence | Array Target ID | Primer Location (hg38) | Size (bp) |
| | | | | | |
| External | cg054\_Ext\_Tail\_FM | CATCTGCCTGAGTATGAACGAAGAGATAGCGGCGG | cg05491001 | chr4:61,200,782-61,200,860 | 79 |
| External | cg054\_Ext\_Tail\_RM | CTTGATGTCAGTTAGGACGACCGAACCGAACAACTA | | | |
| Target | cg054\_M\_Probe | CGACCCCGAACTCTACGC | | | |
| | | | | | |
| External | SCG3\_Ext\_Tail\_FM | CATCTGCCTGAGTATGAATGTTGGAGTTATTGATGTTATT | cg01908954 | chr15:51,681,379-51,681,484 | 106 |
| External | SCG3\_Ext\_Tail\_RM | CTTGATGTCAGTTAGGACCAAAACATAACTTTACTCTCACTCC | | | |
| Target | SCG3\_M\_Probe | AACGCATACTCCATACACGC | | | |
| | | | | | |
| External | ACTB\_Ext\_Tail\_F | CATCTGCCTGAGTATGAAGTTTTTATTTATTTAGGAAGGAAG | | chr7:5,528,269-5,528,346 | 78 |
| External | ACTB\_Ext\_Tail\_R | CTTGATGTCAGTTAGGACAAATCATCACCATTAACAATAAAC | | | |
| Target | ACTB\_Probe | CTACCCTAAAACACTCTTCC | | | |
| | | | | | |
| Target | Tail\_Internal\_F | CATCTGCCTGAGTATGAA | | | |
| Target | Tail\_Internal\_R | CTTGATGTCAGTTAGGAC | | | |
